# Bridging Global Gaps in Cardiovascular Health: Catalyzing Action

**DOI:** 10.1016/j.jacadv.2024.101436

**Published:** 2024-12-26

**Authors:** Sadeer Al-Kindi, Rajesh Vedanthan, Dipti Itchhaporia

**Figure:**
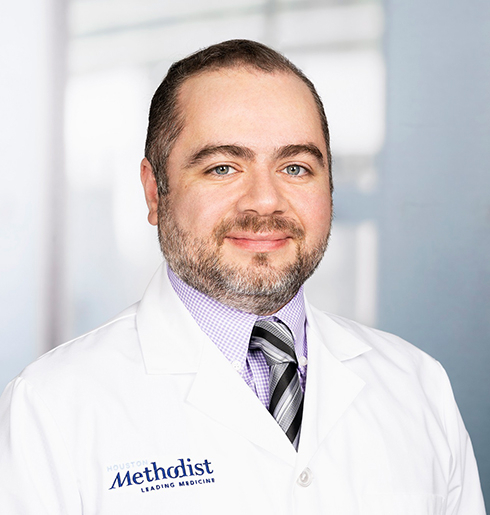

**Figure:**
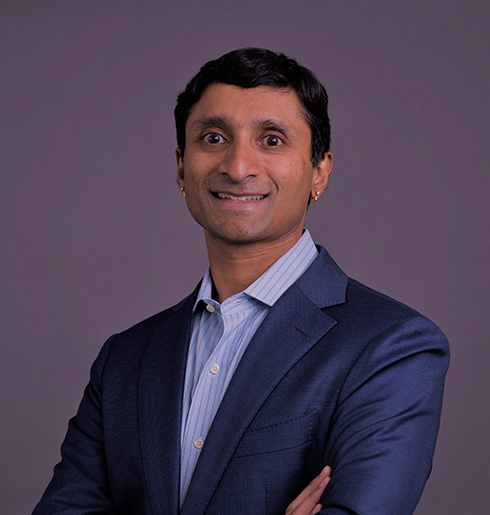

**Figure:**
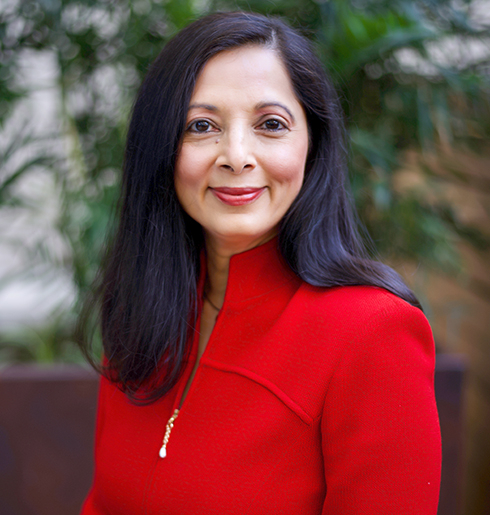

As we step into a new era of global cardiovascular health, this special issue of *JACC: Advances* presents a series of papers offering in-depth insights into the global burden of cardiovascular disease (CVD) and the ongoing inequities that affect regions, populations, and health systems. This issue highlights both the distinct challenges and the innovative strides being made in promoting cardiovascular health worldwide. As the guest editors for this special issue on global CV health, we are proud to introduce contributions that emphasize the urgency of addressing these disparities, highlight the potential for impactful international collaboration on systemic change, and pave the way toward actionable solutions.

This issue further explores the persistent challenge of rheumatic heart disease (RHD) in Africa, a condition that continues to impose a disproportionate burden on low- and middle-income countries (LMICs). In their review, “RHD Burden in Africa and the Need to Build Robust Infrastructure for Effective Control,”^1^ Aliyu et al. address the critical gaps in health infrastructure, emphasizing the need for targeted prevention and management strategies to combat RHD. This theme is further built on by the expert panel led by Bamba Gaye,^2^ in which they advocate collaborative research and intervention strategies through the Alliance for Medical Research in Africa. Together, these contributions underscore the urgent need for global health efforts to prioritize both treatment and prevention of CVD through stronger local and international partnerships.

Nonrheumatic valvular heart disease continues to be a critical global health issue, especially in LMICs. In their analysis of valvular heart disease-related mortality, Hibino et al^3^ reveal stark outcome disparities between middle- and high-income countries. The study by Bhatti et al.^4^ explores solutions to these disparities by advocating for the innovative, multidisciplinary heart team approach at the NICVD Valve Center to optimize valvular disease care in LMICs. The editorial, “Valvular Heart Disease: Geography is Destiny!” by Bakaeen et al,^5^ illustrates how access to care profoundly influences outcomes, with geography often dictating the prognosis for those with severe valvular disease.

Health inequities also extend into specialized areas of CV care. Keva Garg’s paper^6^ “Global Health Inequities in Electrophysiology Care” examines the geographic disparities in access to advanced electrophysiological interventions, such as atrial fibrillation treatments. The study by Frimodt-Møller et al^7^ on ethnic differences in AF in the United Kingdom reveals significant gaps in care, even within resource-rich settings. Similarly, research by Tereshchenko et al^8^ on electrical heterogeneity among Hispanic populations underscores the importance of understanding ethnic disparities in rhythm disorders. In an editorial commentary, Tamirisa and Al-Khatib^9^ emphasize that although these disparities are well-documented, the underlying mechanisms remain unclear, calling for further investigation into genetic, social, and environmental factors.

Heart failure and cardiomyopathies in resource-limited settings are central themes in this issue, highlighted by Kraus et al^10^ via a multicenter pilot study in Southern Africa, “Etiology and Phenotypes of Cardiomyopathy,” which provides insightful data on the burden and progression of heart failure in diverse African populations. The editorial commentary by Bhaskar^11^ continues this conversation by emphasizing the need for expanded access to diagnostic tools and treatments in these regions. The study by Cichowitz et al^12^ on diastolic dysfunction in people with HIV in Tanzania further emphasizes the intersection of infectious diseases and CVD, underscoring how chronic conditions disproportionately impact those in LMICs.

This issue places a spotlight on ischemic heart disease (IHD) outcomes, featuring REACTIV Study in Côte d'Ivoire by Yao et al,^13^ which offers key insights into myocardial infarction outcomes within a sub-Saharan African setting. The study by Romeo et al^14^ on sex disparities in ischemic heart disease mortality in Europe and Dimala’s analysis^15^ of coronary artery disease and MI trends in the United States add a global perspective, illustrating how factors such as gender and geography influence CVD outcomes. Together, these studies underscore the complex, multifactorial nature of CVD, with social determinants of health playing an increasingly critical role.

Innovation in CVD management emerges as a major theme in this issue, particularly in the area of emerging technologies. A scoping review by Osei et al^16^ of electronic health record-based screening algorithms for familial hypercholesterolemia demonstrates how data-driven approaches can improve the identification and management for high-risk populations. Additionally, the work by Sibindi et al^17^ on implementing a virtual cardiology curriculum in Haiti illustrates the potential of digital health solutions to bridge gaps in cardiovascular education and care delivery in underserved regions.

Cardio-obstetrics is also prominently featured, with the survey by Herrera et al^18^ of general cardiologists in Latin America highlighting the growing importance of pregnancy-related cardiovascular care. The scoping review by Yang et al^19^ on the global disparities in outcomes for pregnant individuals with RHD provides an even more urgent view of the compounded risks faced by this vulnerable group, who often contend with systemic health care challenges along with pre-existing conditions.

Environmental determinants of CVD are a critical focus, as highlighted by Adeoye et al^20^ in their scoping review on the impact of air pollution on cardiovascular health in African populations, offering important insights into how environmental factors contribute to the global CVD burden. This theme is reinforced by an expert panel on cardiovascular health promotion in Africa, which advocates for a multi-faceted approach that includes both medical interventions and policy-level actions to address the health effects of air pollution, poverty, and other social determinants of health.^2^

In light of the pressing global health challenges presented in this issue of *JACC: Advances*, the need for action is undeniable. The research and reviews featured here provide not only valuable insights but also a call to address the entrenched inequities in cardiovascular care. Moving forward, prioritizing strategies that address both the clinical and systemic barriers faced by populations in low- and middle-income countries is essential. Key steps include building robust health care infrastructure, advancing digital health initiatives, and fostering global collaboration, while ensuring affordable care and minimizing out-of-pocket expenses by patients and families. Additionally, scaling up innovative technologies, such as electronic health record-based screening tools and virtual cardiology education, will be crucial to ensure broader, equitable access to care.

As researchers, clinicians, and policymakers, we hold the responsibility to act on the evidence presented in these studies. To effectively reduce the global burden of CVD and ensure equitable, healthier futures for all,^21^ we must move beyond awareness to achieve tangible progress. We need clear, measurable goals for improving risk factor control and CVD rates, robust investments in health care systems to ensure equity and accessibility, and the use of advanced data analytics and artificial intelligence to optimize interventions and personalize care. Policy initiatives should promote healthier environments, and cross-sector collaborations must align various stakeholders toward common health goals. Let this issue not only reflect our current challenges but also inspire a shift toward scalable solutions that the global health community has been striving for many years.
